# Variable phenotype associated with compound *LDLR* gene mutations in familial hypercholesterolemia patients: Case series and clinical implications

**DOI:** 10.1097/MD.0000000000047768

**Published:** 2026-02-28

**Authors:** Noor Alicezah Mohd Kasim, Yung-An Chua, Siti Hamimah Sheikh Abdul Kadir, Alyaa Al-Khateeb, Aimi Zafira Razman, Sukma Azureen Nazli, Aisyah Kamal, Johanes Dedi Kanchau, Yeow Siong Lee, Mohamed Syarif Mohamed Yassin, Nadeem Qureshi, Hapizah Nawawi, Anis Safura Ramli

**Affiliations:** a Cardiovascular Advancement and Research Excellence Institute (CARE Institute), Universiti Teknologi MARA, Selangor, Malaysia; b Department of Pathology, Faculty of Medicine, Universiti Teknologi MARA, Selangor, Malaysia; c Department of Biochemistry and Molecular Medicine, Faculty of Medicine, Universiti Teknologi MARA, Selangor, Malaysia; d Selayang Baru Health Clinic, Jln Sungai Tua, Taman Selayang, Batu Caves, Selangor, Malaysia; e Department of Primary Care Medicine, Faculty of Medicine, Universiti Teknologi MARA, Selangor, Malaysia; f Centre of Academic Primary Care, School of Medicine, Faculty of Medicine and Health Sciences, University of Nottingham, Nottingham, United Kingdom.

**Keywords:** compound heterozygous, homozygous familial hypercholesterolemia, *LDLR*, pathogenic variant

## Abstract

**Rationale:**

Homozygous familial hypercholesterolemia (HoFH) is a rare inherited disorder with an extremely elevated level of low-density lipoprotein (LDL) cholesterol (LDL-C) and accelerated premature coronary artery disease (PCAD). It is primarily caused by a single pathogenic variant of the LDL receptor (*LDLR*) gene. This report presents 2 rare and unrelated cases of HoFH with compound *LDLR* mutations. These 2 individuals presented with atypical clinical features and demonstrated variable degrees of hypercholesterolemia.

**Patient concerns:**

Case 1 is a 36-year-old Malay woman identified during family cascade screening with a pretreated LDL-C of 8.5 mmol/L and a strong family history of PCAD. Case 2 is a 58-year-old Indian woman discovered to have a pretreated LDL-C of 5.2 mmol/L during routine health screening, without a significant family history of hypercholesterolemia or PCAD. Neither patient demonstrated tendon xanthomas or other lipid stigmata.

**Diagnoses:**

Both patients underwent lipid profiling and targeted next-generation sequencing of FH-related genes (*LDLR*, *APOB*, *PCSK9*, *ABCG5*, and *ABCG8*). Two novel *LDLR* variants were identified in exon 18: c.2548-1_2548delGAinsTC (pathogenic) and c.2556_2557insTCAGTCTGG (p.Leu853Serfs*12; likely pathogenic) and classified according to American College of Medical Genetics and Genomics guidelines. Case 1 was homozygous for both variants, while Case 2 was homozygous for the splice-site variant and heterozygous for the frameshift variant.

**Interventions:**

Both patients received guideline-directed lipid-lowering therapy and ongoing cardiovascular risk management.

**Outcomes:**

Despite biallelic *LDLR* variants, both patients demonstrated relatively milder hypercholesterolemia and absence of classical HoFH stigmata.

**Lessons:**

The *LDLR* variants located in exon 18 affecting the cytoplasmic tail domain may be associated with attenuated clinical expression. Recognition of genotype–phenotype variability is crucial for accurate diagnosis, risk stratification, and individualized management of HoFH.

## 1. Introduction

Familial hypercholesterolemia (FH) is a genetic disorder of lipid metabolism that is caused by inherited pathogenic mutations in the low-density lipoprotein (*LDLR*), apolipoprotein B (*APOB*), and proprotein convertase subtilisin/kexin type 9 (*PCSK9*) genes.^[1–3]^ Monogenic FH is mostly attributed to mutations in *LDLR* causing a more severe phenotype of FH compared to *APOB*. Because of its autosomal dominant mode of inheritance, mutations in 1 allele of the FH genes result in heterozygous FH (HeFH) phenotypes, in contrast mutation involving both alleles of the same gene leads to homozygous FH (HoFH), with the latter being rarer and with a more severe phenotype occurring roughly in 1 in 160,000 to 300,000 individuals.^[4–6]^

For the majority of HoFH mutation-causing alleles, the mutations are within the same gene (usually *LDLR*), and patients are referred to as “true homozygotes.”^[1]^ HoFH patients with the same mutation on each allele are called “simple homozygotes,” while those with different mutations within the same gene are called ‘compound heterozygotes’. Finally, sporadic patients with HoFH have mutation-bearing alleles in 2 different FH genes: the first is almost always within the *LDLR*, while the second is located in one of the other FH-related genes. Such cases are referred to as “double heterozygotes.”^[5]^

## 2. Case presentation

**Figure 1.**
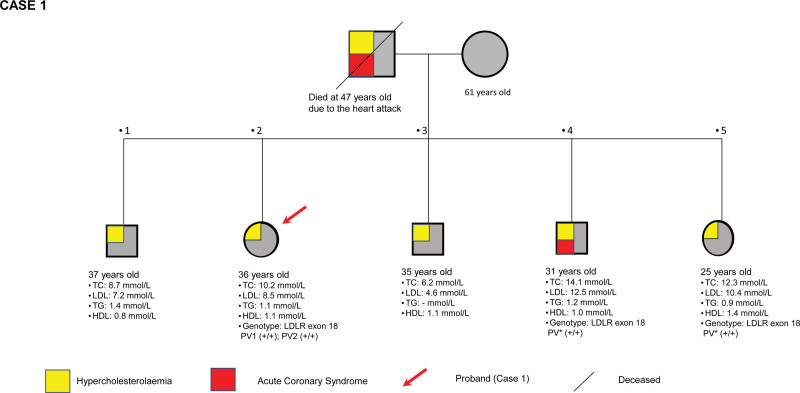
Pedigree of proband’s (case 1) family with pretreated baseline TC, LDL, TG and HDL. HDL = high-density lipoprotein, LDL = low-density lipoprotein, TC = total cholesterol, TG = triglycerides.

Case 1 was a 36-year-old Malay lady who was discovered to have an elevated pretreated baseline serum low-density lipoprotein (LDL-C) at 8.5 mmol/L during a family cascade screening for FH. At that time, her brother, aged 25 years, experienced an acute myocardial infarction and the subsequent lipid profile result showed elevated pretreated baseline LDL-C of 12.1 mmol/L. Surprisingly, there was no visible evidence of lipid stigmata. She was otherwise healthy and did not experience any chest pain or other cardiovascular symptoms. Her father died at 47 years old due to a heart attack. She had a sedentary lifestyle with minimal physical activity. Her family pedigree is shown in Figure 1.

**Figure 2.**
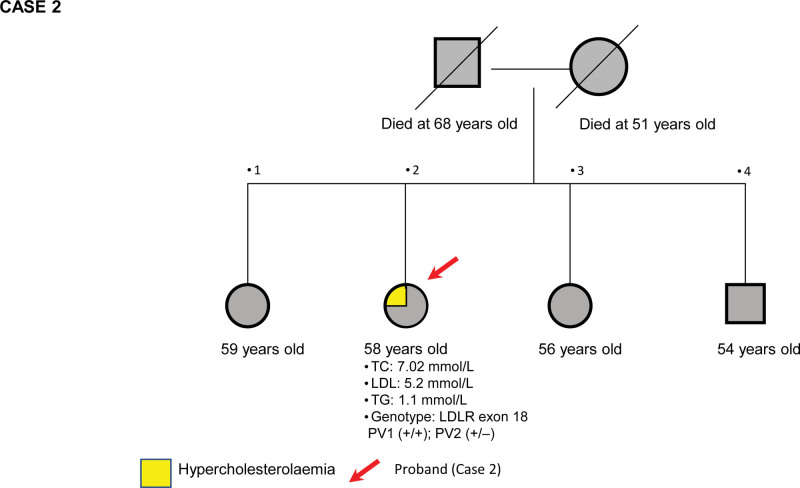
Pedigree of proband’s (case 2) family with pretreated baseline TC, LDL, TG and HDL. HDL = high-density lipoprotein, LDL = low-density lipoprotein, TC = total cholesterol, TG = triglycerides.

Case 2 was a 58-year-old Indian lady who was noted to have an elevated pretreated baseline serum LDL-C of 5.2 mmol/L during a health checkup. She was otherwise healthy, except for hypertension, and had a thyroidectomy operation ten years previously due to hyperthyroidism. She was on 100 ug levothyroxine daily and was clinically and biochemically euthyroid at the time of presentation. Her father and mother died at the ages of 68 and 51, respectively, due to noncardiac causes. All her siblings were healthy and had not yet been screened for hypercholesterolemia. The marital consanguinity status of her parents could not be determined as the patient refused to disclose further details. Her family pedigree is depicted in Figure 2.

Both were then scheduled for genetic analysis for FH candidate genes, which include *LDLR, APOB*, *PCSK9, ABCG5, and ABCG8*. A total of 200 μL of whole blood sample was used to extract the deoxyribonucleic acid (DNA) from the patient’s blood samples using a commercial Lucigen MasterPure™ DNA Purification Kit for Blood Version II (Epicentre, WI, USA) according to the manual instructions of the extraction kit manufacturer. The genetic analysis was conducted through targeted next-generation sequencing (NGS) using the Illumina iSeq100 platform (Illumina, USA). The pathogenicity of the variants was determined according to the American College of Medical Genetics and Genomics (ACMG) guidelines.^[7]^ This analysis covered single-nucleotide variants and small insertions/deletions within the targeted genes. Large *LDLR* rearrangements such as exon-level deletions or duplications were not investigated, as neither Multiplex Ligation-dependent Probe Amplification (MLPA) nor copy number variation detection techniques were employed.

**Figure 3.**
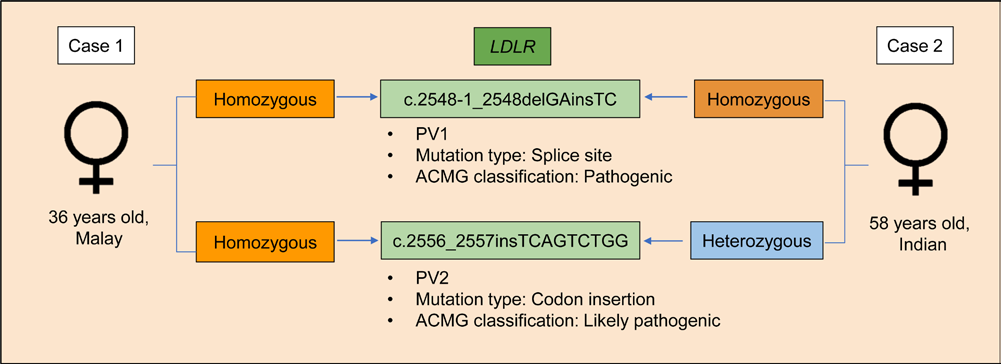
Summary of genetic findings and landscape of case 1 and 2.

In both cases, genetic testing revealed 2 possibly disease-causing variants of *LDLR* located in exon 18, described according to Human Genome Variation Society recommendations using the reference transcript NM_000527.5 and the genome build GRCh37 (hg19). The first variant (PV1), *NM_000527.5:*c.2548-1_2548delGAinsTC; p.(?), which possibly affects the canonical splice-acceptor site and is predicted to disrupt normal exon 18 splicing, leading to a frameshift and premature termination of translation. Following the ACMG Guidelines, this variant with pathogenicity evidence of pathogenic very strong 1 (mutation at splice site), Pathogenic Moderate (PM) 2 (absent in controls) and Pathogenic suPporting (PP) 4 (suggestive patient’s phenotype), is being categorized as “pathogenic.” The second pathogenic variant (PV2), *NM_000527.5:*c.2556_2557insTCAGTCTGG (p.(Leu853Serfs*12)) is a frameshift insertion predicted to introduce a premature stop codon and produce a truncated, nonfunctional LDLR protein. Predicted protein-level consequences were annotated according to the Human Genome Variation Society nomenclature using Mutalyzer (v3.0.4) based on transcript *NM_000527.5*. With the pathogenicity evidence of PM1 (located in the hot spot), PM2 (absent in controls), PM4 (causing protein length change) and PP4 (suggestive patient’s phenotype), this variant was categorized as “likely pathogenic” according to the ACMG guidelines. As neither variants were reported in gnomAD, they were classified as rare and possibly novel. In case 1, the patient was homozygous for both variants, whereas in case 2, the patient was homozygous for PV1 and heterozygous for PV2 in the *LDLR* gene. The genetic findings of both cases are summarized in Figure 3. The younger brother and younger sister of case 1 were already subjected to genetic testing for another study due to their extremely elevated LDL-C levels. Both siblings of case 1 were reported to have a frameshift mutation of c.2553_2556delGATGinsTCT, which affects the normal stop codon and leads to an elongated LDLR protein.

No pathogenic variant (PV) that could be reported in the other FH-related genes (*APOB* and *PCSK9, ABCG5, and ABCG8*). Both variants were supported by high-quality sequencing metrics derived from the variant call format output. The average read depth across the variant loci was approximately 500×, with balanced allele fractions (0.49 for heterozygous) and Phred quality scores above Q20. No strand-bias or low-quality indicators were detected by the variant caller. Although raw alignment files were not available for read-level visualization, the quality metrics strongly support the validity of both variant calls.

Both patients commenced statin therapy with lifestyle modification. However, detailed treatment timelines, subsequent escalation and longitudinal LDL-C responses were not available for documentation.

Written informed consent was obtained from both patients for publication of this case report. The data supporting the findings of this case report are available from the corresponding author upon reasonable request. Due to patient confidentiality restrictions, the datasets are not publicly available.

## 3. Discussion

Mutations in the *LDLR* gene are the most common cause of FH.^[8]^ The *LDLR* gene encodes a protein called the LDL receptor that mediates cholesterol metabolism in humans by binding and internalizing cholesterol transported by LDL. LDL receptors play a crucial role in regulating cholesterol levels by removing LDL from the bloodstream. Hence, mutations in *LDLR* cause the LDL to accumulate instead of being eliminated from the circulation, leading to hypercholesterolemia.

The *LDLR* gene is located at 19p13.2 and comprises 18 exons spanning 45 kilobases (kb). It encodes an 860 amino acid protein that consists of 5 functional domains: N-terminal ligand-binding domain, the epidermal growth factor-precursor homology domain, the O-linked sugars containing domain, the trans-membrane domain, and the C-terminal cytosolic domain.^[1]^ Recently, about 3000 *LDLR* variants were documented in the ClinVar database; however, only a fraction of them were proven to be pathogenic for FH.^[9]^

**Figure 4.**
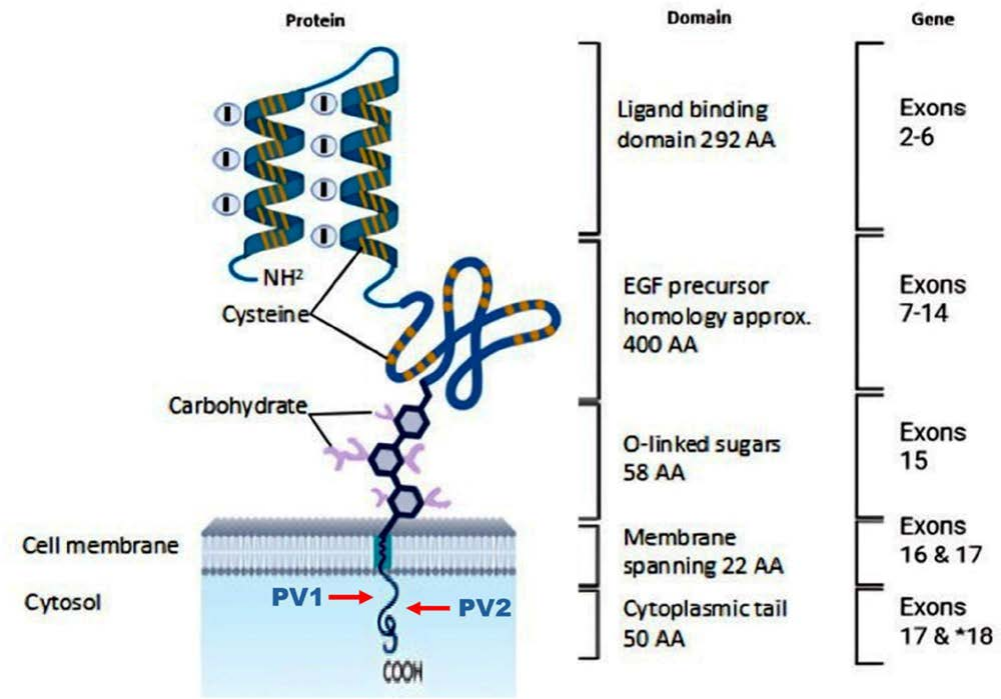
Schematic representation of LDLR protein structure with corresponding exons LDLR structure. LDLR = low-density lipoprotein receptor.

The first PV, c.2548-1_2548delGAinsTC (p.(?)), indicates a deletion of GA with the insertion of TC nucleotides just before codon 2548 in the mRNA transcript. This variant is located at the splice site, which can affect RNA splicing. The second reported variant in these 2 subjects, c.2556_2557insTCAGTCTGG (p.(Leu853Serfs*12)), indicates insertion of the TCAGTCTGG nucleotide sequence at positions 2556 and 2557 of the coding sequence. This type of mutation can lead to changes in the protein sequence encoded by the defective *LDLR*. A very closely related frameshift mutation, c.2553_2556delGATGinsTCT, was previously reported in the same family in our earlier case report,^[10]^ and was discovered in 2 younger siblings of case 1, who both had LDL-C levels >10 mmol/mL, which were even more severe than those of the index individual. This mutation has been putatively identified as a PV. Although all these newly discovered variants were categorically designated as pathogenic and likely pathogenic by ACMG, a functional study is still recommended to confirm their pathogenicity. The domain of the LDLR affected by the 2 variants is illustrated in Figure 4.

Determination of the pathogenicity of FH candidate genes remains a challenge in most molecular studies.^[11]^ This report highlights a rare combination of homozygous compound heterozygous *LDLR* variants involving exon 18 in the cytoplasmic tail domain, which may contribute to the phenotypic variability observed in FH. This report describes 2 rare configurations of *LDLR* mutations identified in clinically diagnosed FH patients. Case 1 is homozygous for both c.2548-1_2548delGAinsTC (p.(?)) and c.2556_2557insTCAGTCTGG (p.(Leu853Serfs*12)) variants in the *LDLR* gene, while Case 2 is homozygous for variant c.2548-1_2548delGAinsTC (p.(?)) and heterozygous for variant c.2556_2557insTCAGTCTGG (p.(Leu853Serfs*12)) in the *LDLR*. Such distinctive mutational patterns are uncommon and expand the growing spectrum of *LDLR* genotypes associated with phenotypic diversity of FH. Interestingly, in both cases, the LDL-C levels were not markedly elevated, in contrast to typical homozygous FH cases, which often present with untreated LDL-C levels of more than 13 mmol/L.^[5,8]^ Moreover, neither patient demonstrated any clinical symptoms of coronary artery disease, such as breathlessness and chest pain, typically observed in HoFH cases with premature coronary artery disease; such symptoms often present during childhood or adolescence, typically within the first or second decade of life.^[12]^ This contrast is notable when compared with a previously reported Malaysian HoFH case that presented with classical severe hypercholesterolemia and early clinical manifestations.^[13,14]^ Furthermore, it is noteworthy that these 2 cases of FH with compound mutations, including homozygous and heterozygous mutations in the *LDLR* gene, exhibit an absence of lipid stigmata such as xanthomata and premature corneal arcus, which are classically reported in HoFH patients.

In these cases, despite having 2 mutations at the *LDLR* gene, the clinical manifestations were not profound except for the severely elevated LDL-C levels, which were of <13 mmol/L. This could be explained by the location of the *LDLR* mutation at exon 18, which corresponds to the cytoplasmic tail domain. Mutations in this region are thought to impair receptor internalization while partially preserving ligand-binding capacity, leading to a milder phenotype compared with alterations in other domains of the LDLR protein. Previous studies have similar results showing that cytoplasmic tail mutations retain the ability of LDLR to bind LDL-C but are defective in internalization, resulting in impaired endocytosis.^[15–17]^ While this provides a biologically credible explanation supported by prior reports, in the present study, it remains an inference, as no functional assays were performed to directly confirm the effect of these variants on protein function. Unexpected variations in the phenotypes were observed in both cases. Case 1 is homozygous for PV1 and PV2, whereas Case 2 is homozygous for PV1 and heterozygous for PV2. Notably, Case 1 exhibits more severe clinical phenotypes, supported by the fact that Case 1 has more severe hypercholesterolemia than Case 2 (LDL: 8.5 mmol/L vs 5.2 mmol/L). Aside from that, we hypothesized that the less harmful effect of HoFH observed in these cases may result from a compensatory mechanism. Although the importation rates of cholesterol into cells would theoretically be inhibited and plasma concentration is increased in FH, cells can compensate by synthesizing more cholesterol and also obtain more cholesterol by low-affinity processes as a result of a higher concentration in their surroundings.^[15–17]^

In compensatory mutation, when multiple mutations occur on the same gene, they can become less deleterious or even advantageous to the carrier due to either the recovery of the original gene function or the acquisition of a new function.^[16]^ Hence, the functional analysis of these novel *LDLR* variants is recommended to comprehend their pathogenicity better and to determine the best feasible treatment strategies in clinical practice. Future studies, including in vitro functional assays, are essential to confirm the pathogenicity and mechanistic impact of these *LDLR* variants, and potential collaborative efforts are being considered to pursue such analyses. Although targeted NGS successfully detected small-scale variants within *LDLR*, large genomic rearrangements such as exon-level deletions or duplications were not detected due to the technical limitation of the NGS platform. Future work incorporating MLPA or copy number variation analysis would be valuable to exclude the presence of large *LDLR* rearrangements and to provide a more comprehensive genetic assessment. While Sanger sequencing and IGV-based read visualization were not performed due to resource constraints, both *LDLR* variants were identified independently in 2 unrelated patients using high-depth targeted NGS (>500 × coverage). The sequencing quality metrics, including read depth, genotype quality, and strand-bias parameters, supported the reliability of these indel calls within exon 18, which is known to be prone to alignment artifacts. Despite the absence of Sanger confirmation, the robust NGS coverage and quality indicators support the reliability of these findings. However, the cis/trans phasing of the affected alleles could not be determined as the short-read NGS used in this study lacks sufficient read length. Future studies employing long-read or linked-read sequencing approaches should be incorporated to provide more accurate phasing information.

Early diagnosis and treatment of HoFH is of great importance. While genetic testing can confirm the diagnosis of HoFH, clinicians should be aware that in certain cases, genetic confirmation remains inconclusive.^[6]^ Plasma levels of LDL-C should not be the only criterion for FH diagnosis, given emerging thoughts that the genetic diversity of HoFH translates to phenotypic variability needs to be considered more than previously thought.

In this study, genetic testing was not performed for all family members, which restricts comprehensive pedigree analysis. Consanguineous marriage may also increase the likelihood of homozygous disease-causing variants,^[6]^ but regrettably remained unconfirmed in case 2 due to the patient’s refusal to disclose the information. In addition, functional validation of the identified *LDLR* variants has not yet been undertaken; therefore, their precise pathogenic mechanisms remain to be clarified. Future studies could include in vitro assays such as LDL uptake or LDLR expression analysis to better understand the functional effects of these variants.

Apart from that, the therapeutic course and longitudinal LDL-C response were not fully available for these 2 patients. Although treatment had been initiated, incomplete records restricted a more detailed evaluation of how the identified variants may have influenced lipid-lowering outcomes over time. A comprehensive follow-up would enrich future understanding of the clinical impact of these novel *LDLR* variants.

## 4. Conclusions

Two patients were reported to be clinically diagnosed with FH and were found to possess rare compound novel pathogenic and likely PVs in the *LDLR* for the first time with a combination of homozygous and heterozygous variants that may lead to variable, nonsevere clinical presentations of FH. The presence of compound variants can result in variable degrees of hypercholesterolemia and clinical presentations among patients with FH, reinforcing that distinct phenotypes may be linked to specific types and combinations of gene variants.

## Acknowledgments

We would like to acknowledge Higher Institution Centre of Excellence (HICoE) research grant 600-RMC/MOHE HICoE CARE-I 5/3 (01/2025) awarded to the Cardiovascular Advancement and Research Excellence Institute (CARE Institute), Universiti Teknologi MARA. Special thanks to the Director General of Health in Malaysia for his permission to publish this article. We would also like to express our gratitude and appreciation to the entire staff of the Selayang Baru Health Clinic for their assistance and support with this study.

## Author contributions

**Conceptualization:** Nadeem Qureshi, Hapizah Nawawi, Anis Safura Ramli.

**Data curation:** Aisyah Kamal, Johanes Dedi Kanchau.

**Formal analysis:** Siti Hamimah Sheikh Abdul Kadir, Alyaa Al-Khateeb, Aimi Zafira Razman, Sukma Azureen Nazli.

**Funding acquisition:** Nadeem Qureshi, Hapizah Nawawi, Anis Safura Ramli.

**Investigation:** Aisyah Kamal, Johanes Dedi Kanchau, Yeow Siong Lee.

**Methodology:** Nadeem Qureshi, Hapizah Nawawi, Anis Safura Ramli.

**Supervision:** Siti Hamimah Sheikh Abdul Kadir, Alyaa Al-Khateeb.

**Validation:** Noor Alicezah Mohd Kasim, Hapizah Nawawi.

**Writing – original draft:** Noor Alicezah Mohd Kasim.

**Writing – review & editing:** Noor Alicezah Mohd Kasim, Alyaa Al-Khateeb, Yung-An Chua, Siti Hamimah Sheikh Abdul Kadir, Mohamed Syarif Mohamed Yassin, Hapizah Nawawi, Anis Safura Ramli.
